# GESDB: a platform of simulation resources for genetic epidemiology studies

**DOI:** 10.1093/database/baw082

**Published:** 2016-05-30

**Authors:** Po-Ju Yao, Ren-Hua Chung

**Affiliations:** Division of Biostatistics and Bioinformatics, Institute of Population Health Sciences, National Health Research Institutes, Zhunan, Taiwan

## Abstract

Computer simulations are routinely conducted to evaluate new statistical methods, to compare the properties among different methods, and to mimic the observed data in genetic epidemiology studies. Conducting simulation studies can become a complicated task as several challenges can occur, such as the selection of an appropriate simulation tool and the specification of parameters in the simulation model. Although abundant simulated data have been generated for human genetic research, currently there is no public database designed specifically as a repository for these simulated data. With the lack of such a database, for similar studies, similar simulations may have been repeated, which resulted in redundant work. Thus, we created an online platform, the Genetic Epidemiology Simulation Database (GESDB), for simulation data sharing and discussion of simulation techniques for genetic epidemiology studies. GESDB consists of a database for storing simulation scripts, simulated data and documentation from published articles as well as a discussion forum, which provides a platform for discussion of the simulated data and exchanging simulation ideas. Moreover, summary statistics such as the simulation tools that are most commonly used and datasets that are most frequently downloaded are provided. The statistics will be informative for researchers to choose an appropriate simulation tool or select a common dataset for method comparisons. GESDB can be accessed at http://gesdb.nhri.org.tw.

**Database URL:** http://gesdb.nhri.org.tw

## Introduction

Computer simulations are routinely conducted in genetic epidemiology studies. For example, when a new statistical method is developed to test associations between genetic variants and a disease, it is important to evaluate the type I error rates for the method and compare the power of the method with other existing methods under different scenarios. Simulation studies are also important to evaluate the study design, such as case-control or family-based design, and to calculate the numbers of samples required to achieve reasonable power when planning a genetic epidemiology study. Because of the complicated structures in human genomes and disease models, simulating realistic genetic variants and trait values can be challenging.

A group consisting of population geneticists, genetic epidemiologists and computational scientists addressed several current and emerging challenges and opportunities in genetic simulation studies in the ‘Genetic Simulation Tools for Post-Genome Wide Association Studies of Complex Diseases’ workshop held at the National Institutes of Health in Bethesda, Maryland on 11–12 March 2014 (1). One of the challenges that was addressed is that researchers may have difficulties in choosing an appropriate simulation tool from a large number of existing tools. For example, the Genetic Simulation Resources (GSRs) website (2) has collected >100 genetic data simulation tools, and each tool has unique properties; however, some tools also share common features. Because of the difficulties in choosing an appropriate simulation tool, the researchers ultimately developed their own tools that had functions overlapping those of the existing tools, which resulted in redundant work (3). Another challenge is that simulated data for a certain study may be generated in favor of the assumptions for the statistical models developed in the study. This could lead to unfair comparisons of the method with other methods. One of the solutions is to create benchmark simulation datasets with detailed documentation for the simulation procedures so that the datasets can become standards for method comparisons (1, 4). The opportunities discussed by the group included the creation of a server for sharing genetic simulation data, identification of common datasets for method comparisons, and encouragement of making simulated datasets publicly available.

In response to the challenges and opportunities addressed above, we created the Genetic Epidemiology Simulation Database (GESDB). The platform consists of a multi-functional website with friendly web interfaces, an FTP server, and a database server. The platform was designed as a repository for simulated datasets generated from published articles or articles under peer review related to genetic epidemiology studies. GESDB has two important features. The first is that each dataset on GESDB can be voted on by the user, and the other is that summary statistics, such as the datasets with the most votes, the most frequently downloaded datasets, and the most frequently used simulation tools, are reported on the main page of GESDB. The summary statistics will be informative to help users select an appropriate simulation tool and a common dataset for method comparisons.

## Methods

### Architecture of GESDB

**Figure 1.**
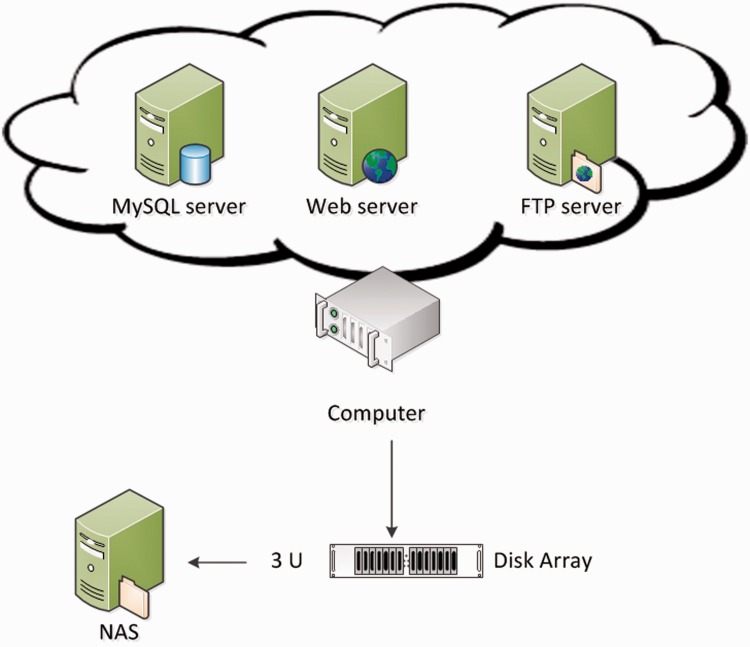
The hardware architecture of GESDB.

Figure 1 shows the hardware architecture of GESDB. The hardware supporting GESDB includes a server-level computer, equipped with an Intel XEON quad-core 2.4 GHz CPU and 96 GB of memory, where the computer is connected by a disk array (with a storage of 50 TB) and a Network Attached Storage (NAS) system with an equal amount of storage to the disk array. The redundant array of independent disks four technique was applied to the disk array as a backup mechanism to protect the data in case of disk failure. The data are copied weekly to the NAS system, which serves as a secondary backup mechanism for the data in the disk arrays. The Web, FTP and MySQL servers were set up on the computer.

### Web server

A person who registers on GESDB and deposits their simulated datasets into the database is referred to as the author, while a person who registers on GESDB and downloads the datasets from the database is referred to as the user. Friendly web user interfaces (UIs) were created for the author and the user on GESDB. The interfaces were tested by four internal and two external users and modified based on their feedback. The author uses an information form to specify the properties of the datasets. The information form collects some basic information about the datasets, including a general description of the data (e.g. study design and types of data) as well as a more detailed survey of the datasets (e.g. the tools and scripts used to generate the data and technical notes for generating the data). Datasets such as simulated raw data, scripts, and any other related files are uploaded to GESDB via the FTP server by the author. The datasets are then classified by the author on the web UI by adding the files to the five categories defined by GESDB. The five categories include ‘Readme’, ‘Scripts’, ‘Result data’, ‘Raw data’ and ‘Other’. The ‘Readme’ category includes documentation such as a description of the simulation steps, while simulation scripts are classified as ‘Scripts’. The results such as type I error rates and power are classified as ‘Result data’, while ‘Raw data’ refers to the simulated raw data. Other file formats are also accepted by GESDB, such as presentation slides and links to published articles, and these are classified as falling into the ‘Other’ category.

The user can search the datasets by data attributes (e.g. article title, keywords and author names) on the web UI and then use the FTP server to download the datasets. The user can also leave comments and vote for a dataset on the web UI. A discussion forum is also hosted on the Web server. The forum provides a platform for questions and answers between the authors and users. On the main page of the web server of GESDB, summary statistics such as the most frequently downloaded datasets, the most frequently used simulation tools, the datasets with the most votes and the most viewed datasets are provided.

### MySQL server

Data attributes from the information form are saved in the MySQL database, and queries sent from the Web server are processed by the MySQL server. The author and user profiles, votes, paths to the files uploaded by the authors on the FTP server, summary statistics and forum discussions are also saved in the MySQL database.

### FTP server

The FTP server handles downloading and uploading the data. Any user can download the data freely via the FTP server. Currently the author can upload files of a maximum size of 50 GB for each study. A folder is created for each author on the FTP server, and the author can create subfolders for different studies. Considering the current storage of 50 TB in the disk array, GESDB will be able to accommodate data from ∼1000 studies. However, because the size of the data for many studies may be significantly <50 GB, we expect that the actual number of studies that GESDB can host will be >1000.

At present, data on GESDB come from datasets deposited by the author, replicated datasets generated by our group and curated web links to other websites consisting of simulated datasets. We selected articles that have clear descriptions of the simulation procedures and followed these procedures to generate replicated datasets. The curated web links were created by our group by a web search to identify websites that contain simulated data for genetic epidemiology studies, and the web links instead of simulation data are saved in GESDB. The websites containing simulated data are usually those generated by the authors of various published articles. Our curators regularly check the web links once per month to ensure that the links are still valid and they will update the database if there are changes of the links from the authors’ websites. The datasets deposited by the author or the replicated datasets generated by our group are under the creative commons (https://creativecommons.org) BY-SA license, which allows licensees to use the datasets if the author is credited (i.e. the author’s article is cited) and allows licensees to distribute derivative works under a license identical to the license. The usage for the datasets hosted on the author’s websites, to which GESDB has linked, is regulated by the author. Each dataset in GESDB is assigned a unique identifier, which can be cited when the dataset is used for other studies.

## Results

**Figure 2.**
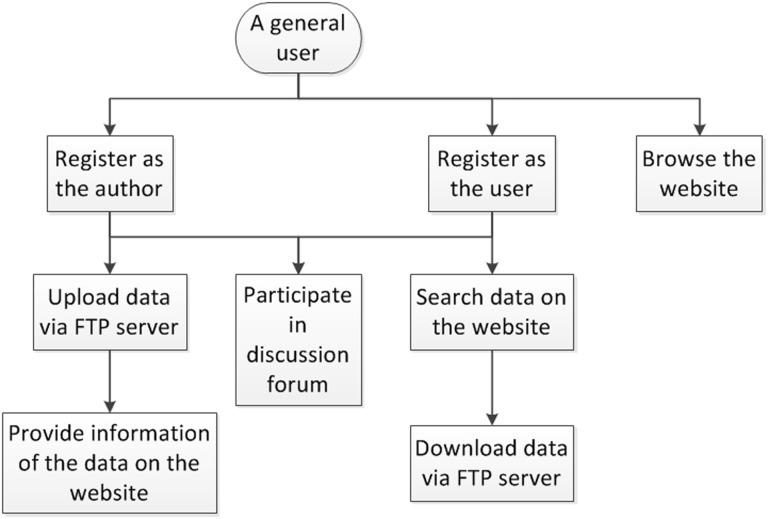
Flowchart for accessing GESDB.

Figure 2 shows the flowchart for a general user to access GESDB. The general user first needs to register on the website to become an author and/or user. After the registration is approved, the author first uploads the datasets via the FTP server. Then, the author fills out the information form on the website to provide information about the uploaded datasets. The user first searches for data on the website and then downloads the data via the FTP server. The author and user can participate in the discussion forum to ask and answer questions. Unregistered users will only be able to browse the datasets and the summary statistics on the website. Note that it is possible for the same person to register as multiple authors or users on GESDB, provided that the person fills different information in the registration form. To avoid repeated votes from the same person, multiple votes from the same IP address will be counted as one vote in the voting system.

The friendly web interfaces were created for the author to upload the data and for the user to search and download the data. Table 1 shows the entries of the information form that the author must fill before uploading the data. Some entries such as the simulated data type and trait type are in the same format as those in GSR. Note that although GESDB aims to host simulated data from published studies, data from articles that are currently under review are also accepted in GESDB. This will provide opportunities for the journal editors or reviewers to assess the simulation scripts and data as part of the review process. Stress tests were performed for both the Web and FTP servers. Both servers functioned normally, assuming that there were 100 simultaneous users who performed regular tasks including web browsing, searching, uploading and downloading the data.

**Table 1.** Information form for the author

Entry	Example	Description
Journal name	*The American Journal of Human Genetics*	The name of the journal where the article is published. Fill in ‘Under review’ for unpublished articles.
Year	2011	The year when the article was published. Fill in the current year for unpublished articles.
Article title	Rare-variant association testing for sequencing data with the sequence kernel association test	The title of the article.
Author	Michael C. Wu, Seunggeun Lee, Tianxi Cai, Michael Boehnke, Xihong Lin	List of author names in the article
Keywords	NA	Keywords in the article
Simulated data type	Sequence	Genotype or sequence
Simulation tool name	SeqSIMLA2^a^	Name(s) of simulation tools used to generate the data
Certification	NA	Certification for the simulation tool, such as GSR certification
Sample type	Case-control	Random or independent; sibpairs, trios and nuclear families; extended or complete pedigrees; case-control; longitudinal
Trait type	Multiple	Binary or qualitative; quantitative; multiple
Determinants of the trait	Multiple genetic markers	Single genetic marker; multiple genetic markers; sex-linked; gene-gene interaction; environmental factors; gene-environment interaction
Brief description of the uploaded data	We followed the descriptions in ‘Numerical Experiments and Simulation’ in the SKAT article (Wu *et al.*, 2011) to simulate the data used for ‘Type 1 Error Simulation’ and ‘Empirical power Simulation’ in the article. The datasets for type 1 error rates were simulated using the regular SeqSIMLA2. The datasets for the power were simulated using a modification of SeqSIMLA2, which can be downloaded as SeqSIMLA_SKATpower in the Script.	A brief description of the uploaded data

a Note that the simulated data used in the original article were generated with the tool developed by the article authors. The datasets on GESDB were the replicated datasets generated by our group using SeqSIMLA2 (12).

The numbers of views as well as votes and comments from users are reported for each dataset on GESDB. On the main page, GESDB reports the summary statistics, including the most frequently used tools, the most frequently downloaded datasets, the most viewed datasets, the datasets with the most votes, and the most viewed and voted posts in the discussion forum. The summary statistics will be informative for other simulation studies, such as choosing a simulation tool that has been widely adopted in the research community. Moreover, the most frequently downloaded datasets may become benchmark datasets for method comparisons. Finally, the forum provides an important communication platform for exchanging simulation strategies and for discussing the simulated data.

## Discussion and Conclusions

Table 2 shows the comparisons between GESDB and two other popular public data repositories, Dryad and figshare. Dryad and figshare are open for the general research community, while GESDB is designed specifically for simulations in genetic epidemiology studies. In terms of hosting genetic simulation data, GESDB has several advantages over these two repositories. GESDB provides a larger free storage space per study (i.e. 50 GB), considering that simulated data are generally large, when compared with the 20 GB free space offered by figshare and the 20 GB space for $120 US dollars offered by Dryad. User statistics such as the number of views and downloads for a dataset are provided for all three repositories, while voting statistics for datasets are uniquely provided by GESDB. Moreover, several crucial summary statistics are also uniquely provided by GESDB, such as the datasets receiving the most votes and the most frequently used simulation tools. These statistics will help eliminate difficulties faced by the user in choosing an appropriate simulation tool and will help researchers identify common datasets for method comparisons. Moreover, a discussion forum is provided by GESDB, making GESDB not only a data repository but also a platform for exchanging simulation strategies.

**Table 2.** Comparison between GESDB and other public data repositories

	GESDB	Dryad	figshare
Data type	Any files related to genetic simulations	Any	Any
Targeted research field	Genetic epidemiology	General	General
Space limit	50 GB free space per study	$120 for the first 20 GB and $50 for each additional 10 GB	20 GB free space
Statistics for each dataset			
Number of views	Yes	Yes	Yes
Number of downloads	Yes	Yes	Yes
Number of votes^a^	Yes	No	No
Summary statistics			
Most frequently downloaded data	Yes	Yes	No
Most viewed data	Yes	No	Yes
Most voted data	Yes	No	No
Most frequently used tools	Yes	No	No
User comment^b^	Yes	No	Yes
Share on social media	No	Yes	Yes
Discussion forum	Yes	No	No
Unique identifier	Yes (GESDB^c^)	Yes (DOI^d^)	Yes (DOI)

a Number of votes given by users.

b Whether users can leave comments on the dataset.

c The identifier is self-defined by GESDB.

d Digital object identifier.

GSR mainly serves as a catalogue of existing genetic simulation tools. Another website, OMICtools (5), constructs a catalog that covers a broader range of tools related to omic data analysis when compared with GSR; however, relatively fewer tools for genetic simulations are collected in OMICtools. The user can search and compare tools on GSR based on different features of the tools, such as simulation method, input and output types and the type of traits. GSR provides certification for a simulation tool based on whether the tool is publicly accessible, is well documented, has been successfully applied to genetic epidemiology studies, and is actively supported by the developers. Because the GSR certification criteria were defined on the basis of the discussions by the experts in the field (1), it is expected that this type of certification will become the norm for genetic simulation software development. Moreover, similar to the purpose of the summary statistics on GESDB, the certification will help the user to determine the most appropriate simulation tools. When compared with GSR, the major advantage of GESDB is that a data repository with simulation data and scripts is included, which will prevent redundant work if the same simulation study is considered by the user and will facilitate statistical method comparisons. Therefore, GESDB can be a complementary resource to GSR. That is, the user can identify an appropriate simulation tool on GSR, and with this information, the user can search and download the datasets simulated by the tool on GESDB.

At present, the simulated data deposited to GESDB are expected to be generated by the author’s local computing resources and uploaded to GESDB via the FTP server. As discussed by Chen *et al.* (1), a genetic simulation server with common application program interfaces (APIs) to different simulation tools would be helpful for the authors to directly simulate data on the server. Such a server would have several advantages. For example, the server would reduce the local computing burden for the author. APIs would also allow for communication among different simulation tools, and modules of common functions such as the generation of sequencing errors could be developed based on the APIs. Moreover, the data simulated on the server would be available for both authors and users and could be stored for later analyses including the selection of benchmark datasets. Furthermore, it would be easier for users to compare results as all analyses would be stored on the server. However, as recognized by Chen *et al.* (1), several challenges still exist, including the creation of an ontology for genetic simulation to develop the APIs, maintenance and storage costs, computing resources and intellectual property issues. The creation of an ontology can be based on other related works such as HuPSON (6), an ontology for simulations in human physiology, but will require more discussion among the genetic simulation community. Moreover, creating a cluster of a large number of computing nodes that fulfill the computing demand from the authors will require a significant amount of funding for purchasing and supporting the hardware. Before these challenges can be resolved, GESDB, which has some common advantages with the proposed server including the storage of simulated datasets and the selection of benchmark datasets, is useful as a genetic simulation resource for the genetic simulation community.

As discussed in Chen *et al.* (1), the cancer intervention and surveillance modeling network (CISNET) group has developed standardized model documentation to facilitate the comparison of simulation or analytical models related to cancer interventions (7). In other research fields, guidelines for reporting simulation studies have also been developed. For example, the Minimum Information About a Simulation Experiment (MIASE) (8), proposed by a group of experts in the field of systems biology where simulations are routinely performed, defines the minimum requirements for describing a simulation experiment. The MIASE guidelines include rules such as a clear description of each simulation model, a precise description of the simulation steps, and the availability to obtain numerical results. Languages such as SED-ML (9) or SBRML (10) have also been developed to formally describe the guidelines for reporting studies in the field of systems biology, which can facilitate the exchange of data between users. Some of the MIASE guidelines are also applicable to genetic simulation studies, while some rules that are more specific to the field of genetic simulation, such as the minimum requirement to validate a statistical method, may be required. Similar to CISNET and MIASE, developing a guideline for describing a genetic simulation experiment will require discussion from a consortia of experts in the field. Other than the pre-specified entries in the information form shown in Table 1, GESDB is also flexible in terms of adding new entries. Therefore, the authors are encouraged to follow similar guidelines as MIASE to provide further detailed information for the data on GESDB. Furthermore, if a guideline for reporting a simulation experiment is developed by the genetic simulation community, it will be incorporated as a required entry on the information form on GESDB.

Another database management system, SEEK (11), was also developed for data and model sharing in systems biology. GESDB and SEEK can both store heterogeneous datasets such as raw simulated data, documentation (e.g. simulation steps), simulation models, and publication information. SEEK provides versioning of datasets and data can be restricted for access to specific users, while the datasets on GESDB are publicly available to all registered users. However, voting and summary statistics are not provided in SEEK. If the simulation models follow the systems biology markup language format (13), SEEK allows for direct simulations on the platform. Direct simulation on a common platform is similar to the concept of the simulation server as discussed in Chen *et al.* (1). Again, this addresses the importance of developing a standard language for simulation models in genetic simulations.

In conclusion, a very useful platform GESDB was created for genetic data simulations. With the information provided by GESDB, it will become straightforward for the user to identify the most appropriate simulation tool. In addition, benchmark datasets can be selected, which can become common datasets for method comparisons. GESDB aims to promote simulation data sharing and improve transparency and efficiency in simulation studies for genetic epidemiology. GESDB is funded by an intramural grant, which was awarded for the period of 2015–19 from the National Health Research Institutes in Taiwan. The 5-year grant will allow us to continue the development of GESDB and to expand its hardware structure. Funding support for GESDB after 2019 will be sought through the same funding agency or another major funding agency, the Ministry of Science and Technology in Taiwan. GESDB can be accessed at http://gesdb.nhri.org.tw.

## Funding

This work was funded by a grant from the National Health Research Institutes (PH-105-PP-10) in Taiwan.

*Conflict of interest*. None declared.

## References

[1] Chen H.S. Hutter C.M. Mechanic L.E (2015) Genetic simulation tools for post-genome wide association studies of complex diseases. Genet. Epidemiol., 39, 11–19. 25371374 10.1002/gepi.21870 PMC4270837

[2] Peng B. Chen H.S. Mechanic L.E (2013) Genetic Simulation Resources: a website for the registration and discovery of genetic data simulators. Bioinformatics, 29, 1101–1102. 23435068 10.1093/bioinformatics/btt094 PMC3624809

[3] Peng B. Chen H.S. Mechanic L.E (2015) Genetic data simulators and their applications: an overview. Genet. Epidemiol., 39, 2–10. 25504286 10.1002/gepi.21876 PMC4804465

[4] Mechanic L.E. Chen H.S. Amos C.I (2012) Next generation analytic tools for large scale genetic epidemiology studies of complex diseases. Genet. Epidemiol., 36, 22–35. 22147673 10.1002/gepi.20652 PMC3368075

[5] Henry V.J. Bandrowski A.E. Pepin A.S (2014) OMICtools: an informative directory for multi-omic data analysis. Database, 2014, pii. 10.1093/database/bau069 PMC4095679 25024350

[6] Gundel M. Younesi E. Malhotra A (2013) HuPSON: the human physiology simulation ontology. J. Biomed. Semantics, 4, 35. 24267822 10.1186/2041-1480-4-35 PMC4177144

[7] Habbema J.D. Schechter C.B. Cronin K.A (2006) Modeling cancer natural history, epidemiology, and control: reflections on the CISNET breast group experience. J. Natl. Cancer Inst. Monogr., 2006, 122–126. 17032902 10.1093/jncimonographs/lgj017

[8] Waltemath D. Adams R. Beard D.A (2011) Minimum Information About a Simulation Experiment (MIASE). PLoS Comput. Biol., 7, e1001122. 21552546 10.1371/journal.pcbi.1001122 PMC3084216

[9] Waltemath D. Adams R. Bergmann F.T (2011) Reproducible computational biology experiments with SED-ML–the Simulation Experiment Description Markup Language. BMC Syst. Biol., 5, 198. 22172142 10.1186/1752-0509-5-198 PMC3292844

[10] Dada J.O. Spasic I. Paton N.W (2010) SBRML: a markup language for associating systems biology data with models. Bioinformatics, 26, 932–938. 20176582 10.1093/bioinformatics/btq069

[11] Wolstencroft K. Owen S. Du Preez F (2011) The SEEK: a platform for sharing data and models in systems biology. Methods Enzymol., 500, 629–655. 21943917 10.1016/B978-0-12-385118-5.00029-3

[12] Hucka M. Finney A. Sauro H.M (2003) The systems biology markup language (SBML): a medium for representation and exchange of biochemical network models. Bioinformatics, 19, 524–531. 12611808 10.1093/bioinformatics/btg015

[13] Chung R.H. Tsai W.Y. Hsieh C.H (2015) SeqSIMLA2: simulating correlated quantitative traits accounting for shared environmental effects in user-specified pedigree structure. Genet. Epidemiol., 39, 20–24. 25250827 10.1002/gepi.21850

